# A Self-Expanding Nitinol Fixation System for Atrial Leadless Pacemakers: Biomechanical Design and Evaluation

**DOI:** 10.3390/bioengineering12050512

**Published:** 2025-05-12

**Authors:** Yu-Tzu Wang, Yu-Sheng Lin, Yu-Wei Lin, Chun-Ming Chang, Lung-Sheng Wu, Chao-Sung Lai, Pao-Hsien Chu

**Affiliations:** 1 Department of Mechanical and Electro-Mechanical Engineering, TamKang University, New Taipei City 251, Taiwan; ytlwh@mail.tku.edu.tw; 2 Department of Cardiology, Chang Gung Memorial Hospital, Chiayi 613, Taiwan; yushuhuang1212@gmail.com (Y.-S.L.); r5278@adm.cgmh.org.tw (L.-S.W.); 3 College of Medicine, Chang Gung University, Taoyuan 333, Taiwan; 4 National Center for Instrumentation Research, National Institutes of Applied Research, Hsinchu 302, Taiwan; lin@narlabs.org.tw (Y.-W.L.); gmp@niar.org.tw (C.-M.C.); 5 Division of Cardiology, Chang Gung Memorial Hospital, Linkou, Taoyuan 333, Taiwan; 6 Institute of Pioneer Semiconductor Innovation, Industry Academia Innovation School, National Yang Ming Chiao Tung University, Hsinchu City 302, Taiwan; cslai@nycu.edu.tw; 7 Institute of Stem Cell and Translational Cancer Research, Chang Gung Memorial Hospital, Taoyuan 333, Taiwan

**Keywords:** leadless pacemaker, left atrial appendage (LAA), nitinol alloy, finite-element analysis, fixture

## Abstract

Atrial leadless pacemakers (ALPMs) offer a minimally invasive solution for patients requiring atrial pacing, but current designs face significant challenges related to fixation stability, perforation risk, and retrievability. This study presents a novel self-expanding nitinol fixation system designed for deployment within the left atrial appendage (LAA), incorporating a flexible adapter for secure pacemaker engagement and retrieval. Finite-element simulations were conducted to assess gravitational displacement across different anatomical orientations, and fixture-expansion behavior was analyzed under various mesh configurations. The pacemaker drop analysis results demonstrated minimal displacement in neutral and upward-tilted LAA models, with increased instability observed in downward-tilted orientations. The fixture-expansion study showed that the 0.2 mm mesh design provided adequate mechanical strength and strain tolerance while maintaining a compact profile. This novel fixation system improves current ALPM limitations by providing stable, retrievable anchoring and favorable biomechanical performance. It may also serve as a dual-function platform for atrial pacing and stroke prevention when integrated with a left atrial appendage (LAA) occluder. These findings support further preclinical validation for clinical translation.

## 1. Introduction

Leadless pacemakers represent a transformative approach in cardiac rhythm management by eliminating transvenous leads and subcutaneous pockets, reducing infection risk and procedural complexity [1,2,3,4,5,6]. Currently, commercially available systems such as the Micra™ (Medtronic, Minneapolis, MN, USA) and Aveir™ VR (Abbott, Chicago, IL, USA) are approved for use in the right ventricle (RV), where they achieve fixation via nitinol tines or active screw-in helix mechanisms. These fixation approaches are effective in the RV due to its relatively thick myocardial wall and stable contractile environment, which minimizes the risks of perforation and dislodgement.

In contrast, the atrial environment poses significant biomechanical challenges for leadless pacing. The right atrial appendage (RAA) features thinner myocardial walls, higher anatomical variability, and more dynamic wall motion influenced by respiration and atrial contraction [7,8,9]. These factors complicate secure long-term fixation using conventional ventricular-derived systems [1,10,11,12]. Moreover, the lack of FDA-approved or CE-marked atrial-specific leadless pacemakers highlights an unmet need for safer, anatomically adaptive atrial fixation systems.

Recent developments, such as the Aveir DR system (Abbott), have introduced dual-chamber leadless pacing using two independent communicating devices, one in the right atrium and one in the right ventricle. The Aveir DR i2i study demonstrated high performance and safety over 12 months, with a 92.8% success rate in atrial capture and sensing and an 88.6% complication-free rate. However, this approach still employs screw-in, or tine-based fixation methods originally designed for the ventricle, and long-term anchoring in the thinner atrial wall remains a critical concern. These findings reinforce the need for improved fixation strategies tailored specifically for atrial deployment [11].

Hirsch et al. recently showed that electrophysiologic studies can help guide atrial-only leadless pacing in select patients with intact atrioventricular conduction. However, fixation in the thin, mobile atrial wall remains challenging, as current devices like the Aveir AR still rely on screw-in mechanisms originally designed for the ventricle. This underscores the need for fixation technologies tailored specifically to atrial anatomy [13].

To address these limitations, this study introduces a novel self-expanding nitinol fixture designed for atrial leadless pacemaker (ALPM) deployment within the left atrial appendage (LAA) [8,14,15,16,17]. The system incorporates a flexible adapter for secure pacemaker engagement and potential retrieval. Through computational modeling and finite-element simulation, we evaluate gravitational displacement across anatomical orientations and biomechanical behavior during fixture expansion. This work supports the development of a dual-function platform capable of providing atrial pacing and stroke prevention within a single device architecture.

## 2. Materials and Methods

### 2.1. Reconstruction of a Left Atrial Appendage (LAA) Model

**Figure 1:**
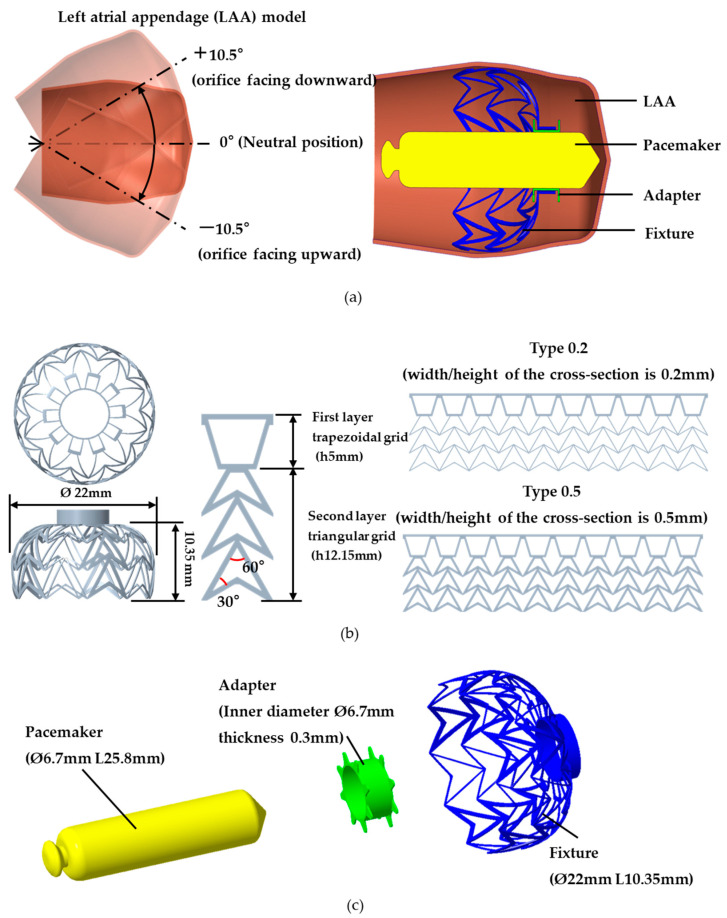
Models used in the analysis: (**a**) overview of the analysis model and LAA orifice orientation; (**b**) fixture geometry and features; (**c**) key dimensional characteristics of the pacemaker, adapter, and fixture.

In this study, a left atrial appendage (LAA) model was developed using computer-aided design (CAD) software (Creo Parametric v8.0, PTC, Needham, MA, USA) based on literature data. The LAA exhibits an enclosed tube structure with an orifice diameter of Ø10.27 mm, a length of 45 mm [8], and a wall thickness of 0.5 mm [14]. Depending on variations in cardiac anatomy, the relative orientation of the LAA concerning the horizontal plane can be classified into three positions: 0° (neutral position), +10.5° (orifice facing downward), and −10.5° (orifice facing upward) (Figure 1a) [18]. These three LAA models can be used for pacemaker drop and fixture-expansion analyses.

### 2.2. Design of the Fixture, Pacemaker, and Adapter Model

The fixture is designed with a structure like an occluder, featuring an umbrella-like shape with an overall cross-sectional diameter of Ø22 mm and a height of 10.35 mm. The grid consists of one layer of trapezoidal grid and three layers of triangular grid (totaling 10 rows). The trapezoidal grid enhances the structural strength of the fixture, while the triangular grid provides flexibility for expansion. Each pillar strut has a square cross-section, with a width/height of either 0.2 mm or 0.5 mm (0.2 type and 0.5 type). The top of the fixture features a circular opening designed to secure both the pacemaker and the adapter. The umbrella-shaped structure has an initial diameter of Ø8 mm before expansion. Upon deployment, the fixture expands to Ø22 mm, ensuring stable fixation within the LAA.

The fixation device developed in this study primarily supports the Medtronic leadless pacemaker (Micra leadless pacemaker, Medtronic, Inc., Minneapolis, MN, USA) [19]. Therefore, for analysis purposes, the pacemaker model was designed to replicate the Medtronic leadless pacemaker, with a diameter of Ø6.7 mm and a length of 25.8 mm. The Medtronic leadless pacemaker features a hook mechanism to secure the pacemaker to the cardiac wall. However, since the fixture will replace this function, the pacemaker model was designed without incorporating the hook structure.

The adapter is intended to be fabricated using highly elastic polymer material, with an inner diameter of Ø6.7 mm and a wall thickness of 0.3 mm (Figure 1a,b). It was designed to securely attach to the pacemaker, facilitating integration with the fixation device (Figure 1c). Additionally, the adapter features acicular features to prevent lateral slippage between the adapter and the pacemaker, ensuring stable positioning.

### 2.3. The Pacemaker Drop Analysis

**Figure 2:**
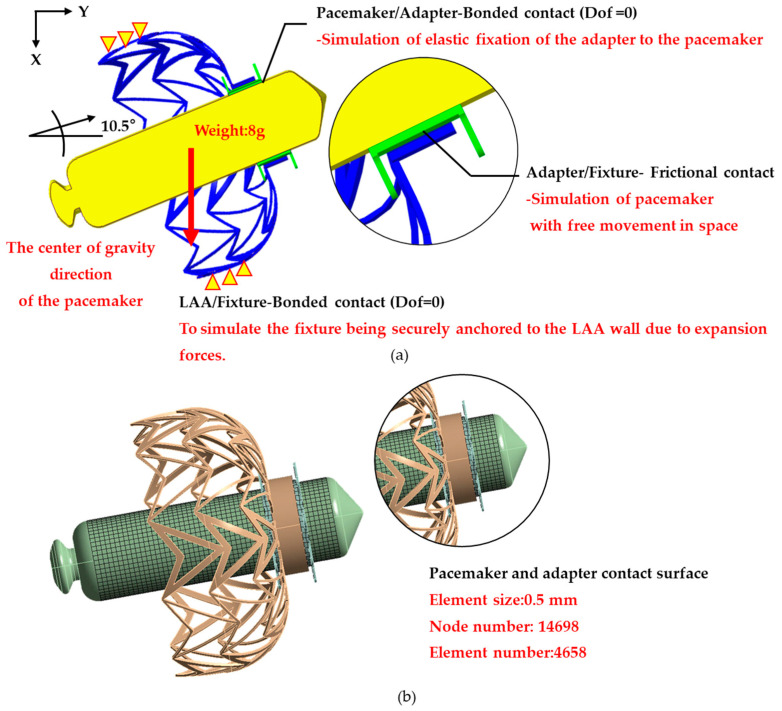
The pacemaker drop analysis model. (**a**) A schematic representation of the contact, loading, and boundary conditions. (**b**) An illustration of the mesh model.

To verify that the designed adapter effectively secures the pacemaker to the fixation mechanism, this study employs finite-element analysis (FEA) to evaluate the impact of gravitational forces on fixation stability. The analysis used the Rigid Dynamics module in ANSYS 2023R2 (ANSYS Inc., Canonsburg, PA, USA). The drop analysis model comprises the fixture, adapter, and pacemaker (Figure 2a). Although the fixture is made of nickel–titanium alloy (Nitinol), it is assumed to be fully expanded in the drop analysis. To simplify the material properties, the fixture was modeled as an elastic material with a Young’s modulus of 60 GPa and a Poisson’s ratio of 0.36. The pacemaker casing, made of titanium alloy [3], is assigned to Young’s modulus of 115 GPa, a Poisson’s ratio of 0.361, and a weight of 8 g. The adapter is assumed to be composed of polylactic acid (PLA), with a Young’s modulus of 2.6 GPa and a Poisson’s ratio of 0.35 [20]. The mesh model utilizes a free mesh at the contact interface between the pacemaker and adapter, with an element size of 0.5 mm. The mesh density and distribution are illustrated in Figure 2b. The adapter-pacemaker interface was defined as a bonded contact, meaning the adapter elastically secures the pacemaker without relative displacement. The fixture–adapter interface is modeled as a frictional contact, with a friction coefficient of 0.3, indicating sliding resistance proportional to the friction coefficient, allowing the two components to separate. The boundary condition assumes the outer surface of the fixture was fixed (Dof = 0), simulating its attachment within the LAA. To simulate the gravitational effect on the pacemaker, a standard Earth gravity load was applied in the pacemaker barycenter of the X-direction, assessing the resulting displacement and confirming whether the fixture and adapter can provide adequate fixation stability.

### 2.4. The Fixture-Expansion Analysis

**Figure 3:**
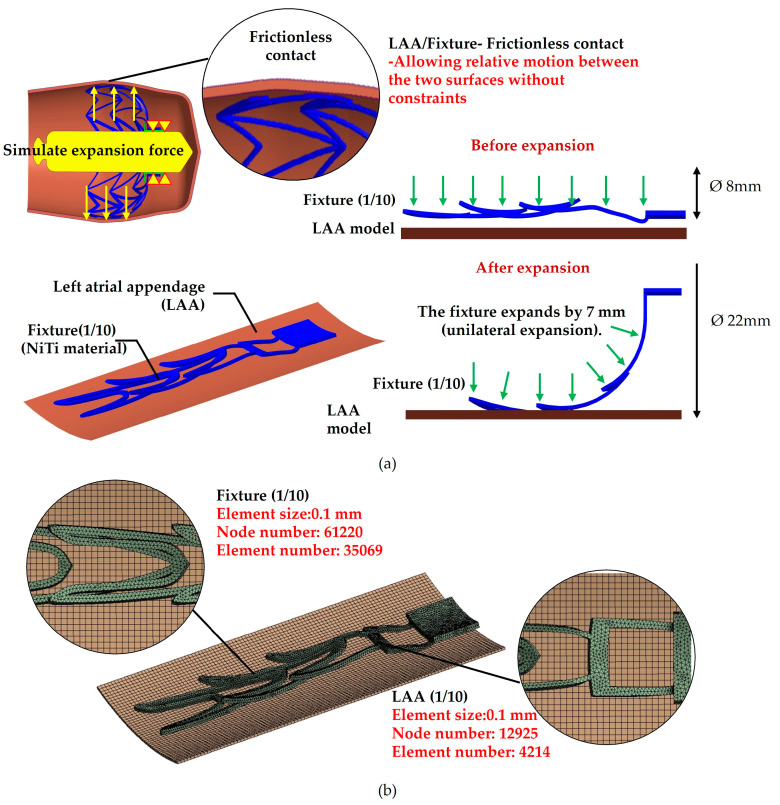
The fixture-expansion analysis model. (**a**) A schematic representation of the contact, loading, and boundary conditions. (**b**) An illustration of the mesh model.

**Figure 4:**
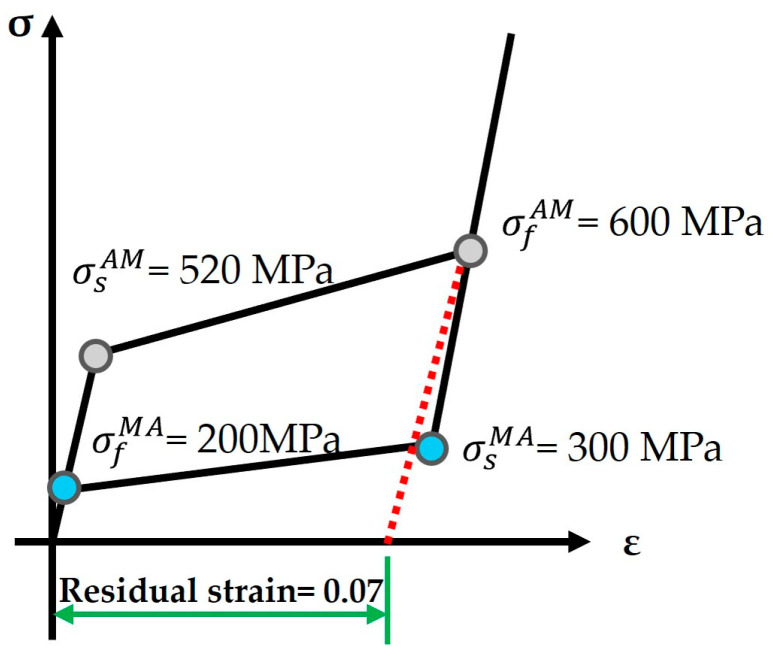
The idealized strain–stress diagram of the superplastic behavior of NiTi alloy.

This study employs fixture-expansion analysis to evaluate the biomechanical behavior of fixation mechanisms with different stent widths when anchored within the LAA. The primary objective is to ensure secure anchoring while minimizing excessive pressure on surrounding tissues. The fixture-expansion analysis model consists of the fixation mechanism and the LAA model, specifically in the +10.5° orientation (orifice facing downward). The fixation mechanism is categorized into two types based on stent width: 0.2 type and 0.5 type. Since the fixation mechanism and LAA model exhibit axial symmetry, the model is simplified to 1/10 of its full geometry to accelerate computational efficiency. The mesh model for the expansion analysis follows the same configuration as the drop analysis, with the mesh density and element distribution illustrated in Figure 3. The fixture is modeled as a shape memory alloy (SMA), exhibiting superelasticity. The idealized stress–strain behavior of superelasticity is shown in Figure 4 [21]. The nickel–titanium (NiTi) alloy properties are defined as follows: Young’s modulus: 60 GPa, Poisson’s ratio is 0.36, Austenite-to-Martensite transformation start stress is 520 MPa, Austenite-to-Martensite transformation end stress is 600 MPa, Martensite-to-Austenite transformation start stress is 300 MPa, Martensite-to-Austenite transformation end stress is 200 MPa, and residual strain is 0.07. Using these parameters, a uniaxial superelastic material model for the fixture was developed to accurately simulate its mechanical response within the LAA.

The material properties of the LAA were simulated using the Mooney–Rivlin model in ANSYS software, which represents the superelastic behavior of the LAA tissue. The Mooney–Rivlin model assumes that the material is isotropic and incompressible, with stress determined based on the derivative of the strain-energy function (*W*). The governing equation for the five-parameter model is given as:

(1) $$\sigma_{ij}=\frac{\partial w}{\partial \varepsilon ij}$$

W = C_10_(Ī_1_ − 3) + C_01_(Ī_2_ − 3) + C_20_(Ī_1_ − 3)^2^ + C_11_(Ī_1_ − 3) (Ī_2_ − 3) + C_02_(Ī_1_ − 3)^2^ (2)

This equation provides an accurate representation of the nonlinear mechanical behavior of the LAA, ensuring realistic simulation results in the expansion analysis.

The parameters C_10_, C_01_, C_20_, C_11_, and C_02_ are material constants, while σ_ij_ represents the Piola–Kirchhoff stress. The strain invariants Ī_1_ and Ī_2_ are computed using the Cauchy–Green deformation tensor. The material parameters used in this study are summarized in Table 1 [22]. When the fixture expands and is in contact with the LAA, the contact condition is defined as frictionless contact, allowing for relative motion between the two surfaces without constraints. For the boundary conditions, the contact surface between the fixture and adapter is fixed (DoF = 0). A standard displacement of 7 mm is applied to the inner surface of the umbrella-shaped structure of the fixture, simulating its expansion from an initial diameter of Ø8 mm to a final diameter of Ø22 mm, thereby securing it within the LAA. Following the simulation, the stress and strain values of the fixture were extracted to evaluate its mechanical response under expansion conditions. The analysis assesses the influence of different stent widths on the fixture’s biomechanical behavior, ensuring optimal anchoring stability while minimizing stress on the surrounding tissue.

## 3. Results

### 3.1. Results of the Pacemaker Drop Analysis

The results of the pacemaker drop analysis indicate that the 0° (neutral) LAA orientation provides a pacemaker displacement of 0.21 mm, while the −10.5° (orifice facing upward) orientation results in a displacement of only 0.058 mm. These orientations offer excellent fixation stability for the pacemaker. However, when the LAA is positioned at +10.5° (orifice facing downward), the pacemaker displacement increases significantly to 0.46 mm (Table 2). This analysis highlights that different LAA orientations present varying risks of pacemaker dislodgement. Although these displacements are relatively small in a clinical setting, they may contribute to long-term fixation challenges. Therefore, optimizing the pacemaker-anchoring mechanism based on patient-specific anatomical variations could help reduce the risk of displacement, particularly in high-risk orientations.

### 3.2. Results of the Fixture-Expansion Force Analysis

**Figure 5:**
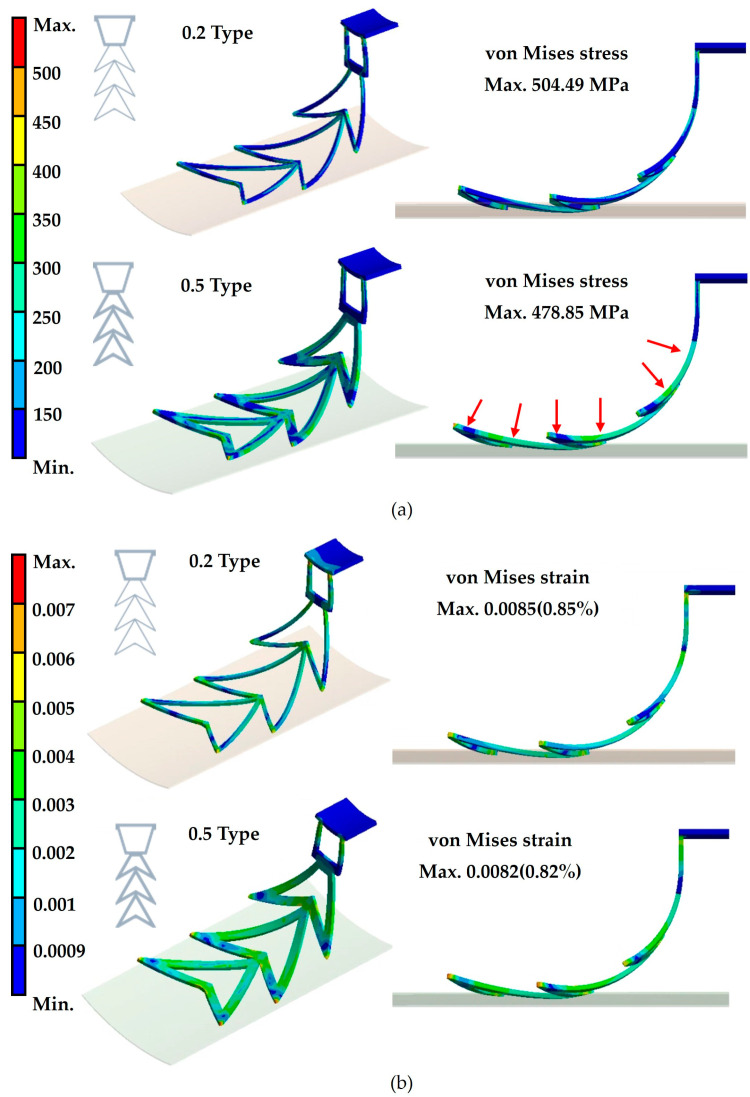
The results of the fixture-expansion force analysis for 0.2-type and 0.5-type fixtures. (**a**) Fixture von Mises stress. (**b**) Fixture von Mises strain.

The results of the fixture-expansion analysis demonstrate that both 0.2-type and 0.5-type fixtures successfully expand from an initial diameter of Ø8 mm to a final diameter of Ø22 mm. This confirms that the fixture’s self-expanding properties enable secure anchoring within the LAA. For the 0.2 type fixture, the von Mises stress is 504.49 MPa, with a corresponding von Mises strain of 0.0085 (0.85%). In contrast, the 0.5 type fixture exhibits a von Mises stress of 478.85 MPa and a von Mises strain of 0.0082 (0.82%) (Figure 5). According to previous studies, NiTi alloy has an ultimate tensile strength of 1133 MPa, and when the strain exceeds 6%, it undergoes a phase transformation to permanent deformation, increasing the risk of permanent deformation [23]. By comparing the analysis results with these reference values, it is evident that both fixture types exhibit stresses well below the ultimate tensile strength of NiTi alloy, and their strain levels remain significantly lower than the 6% threshold. This confirms that neither fixture type is at risk of permanent deformation during expansion. The difference between the 0.2 and 0.5 type fixtures in their von Mises stress. The 0.2 type fixture requires only 478.85 MPa to achieve self-expansion and secure the pacemaker within the LAA. Considering the clinical preference to minimize the volume of implanted devices, this study ultimately selects the 0.2 type fixture as the optimal design, enhancing its adaptability to the dynamic cardiac environment.

## 4. Discussion

This study demonstrates the biomechanical feasibility, safety, and potential clinical impact of a self-expanding nitinol fixture designed to securely anchor ALPMs within the LAA. The pacemaker drop analysis revealed that anatomical orientation significantly affects fixation performance, with downward tilted (+10.5°) configurations posing the highest risk for device migration. These findings support the clinical need for anchoring systems that adapt to individual atrial geometries, improving procedural success and long-term device retention.

The fixture-expansion analysis further validated the design’s structural integrity and mechanical performance. The 0.2 mm strut mesh demonstrated optimal performance, balancing a low-profile design for catheter-based delivery with sufficient expansion force for stable fixation. Despite slightly higher stress values than the 0.5 mm variant, the 0.2 mm design remained within the safe superelastic range of NiTi, confirming its resilience for dynamic cardiac environments and long-term function.

This novel fixture design may address several limitations of existing ALPM technologies [1,7,9,24]. Abbott’s Aveir DR dual-chamber leadless pacemaker system has recently addressed significant market gaps by providing a leadless solution for dual-chamber pacing, expanding to atrial indications, and offering advanced communication and retrievability features [1,10,11,12]. Reducing perforation, dislodgement, and non-retrievability risks can lower complication rates, improve procedural safety, and expand the population eligible for leadless atrial pacing [22,25,26]. The design’s compatibility with minimally invasive catheter-based systems aligns with the current electrophysiological workflow and could shorten procedural times and recovery. Furthermore, its retrievable nature allows for future device upgrades, battery replacement, or troubleshooting without the accumulation of abandoned devices, addressing primary concerns in current leadless systems. The proposed system’s compatibility with standard transcatheter delivery tools may allow for seamless integration into existing electrophysiology workflows, facilitating adoption and reducing the learning curve.

The fixture’s modular design and geometric conformity to the atrial appendage also support integration with LAA occlusion devices. This opens new opportunities for dual-function therapeutic platforms, enabling simultaneous stroke prevention and atrial pacing in patients with atrial fibrillation or high thromboembolic risk. Such a strategy could be particularly valuable in elderly or comorbid populations where procedural efficiency and reduced device burden are critical.

This technology may offer significant cost savings and workflow efficiency by reducing procedural complexity and avoiding transvenous access, especially in aging populations with rising comorbidities. Clinical translation of this technology has the potential to redefine the landscape of atrial pacing by offering a safer, more adaptable, and functionally integrated alternative to existing ALPM solutions. In future study, in vivo studies will be conducted using a suitable large animal model (Porcine Model) to evaluate several key aspects: (1) the stability of fixture anchoring under dynamic cardiac conditions; (2) the long-term biocompatibility and endothelialization response; and (3) the functional performance in terms of both LAA closure and pacing/sensing capability over time. These studies will provide critical insights into device–tissue interaction, healing response, and potential fatigue-related changes under physiological loading.

## 5. Conclusions

This study developed a self-expanding NiTi fixture to enhance the stability and retrievability of atrial leadless pacemakers (ALPMs) in the left atrial appendage (LAA). The pacemaker drop and the fixture-expansion analysis confirm that the design reduces the risks of perforation and dislodgement while maintaining secure fixation. The optimized 0.2-type fixture provides sufficient expansion force without exceeding material limits, ensuring long-term stability.

## Figures and Tables

**Table 1:** Values of Mooney–Rivlin parameters used for the LAA model.

Values of Mooney–Rivlin Parameters Used for the LAA Model		
Parameter	Value	Unit
C_10_	−5.84 × 10^4^	Pa
C_01_	6.34 × 10^4^	
C_20_	1.60 × 10^7^	
C_11_	−3.53 × 10^7^	
C_02_	1.97 × 10^7^	

**Table 2:** Effect of different LAA orientations on pacemaker fixation stability.

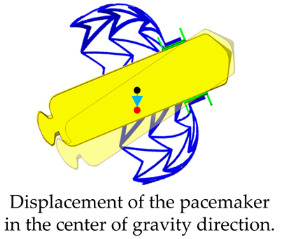	Orientation of the LAA Orifice	Displacement
	0° (neutral)	0.21 mm
	−10.5° (orifice facing upward)	0.058 mm
	+10.5° (orifice facing downward)	0.46 mm

## Data Availability

All data generated during the study can be found in this manuscript.

## Abbreviations

The following abbreviations are used in this manuscript:

ALPMs	Atrial leadless pacemakers
LAA	Left atrial appendage
CAD	computer-aided design
